# Evaluation of accuracy of one-step nucleic acid amplification (OSNA) in diagnosis of lymph node metastases of papillary thyroid carcinoma. Diagnostic study

**DOI:** 10.1016/j.amsu.2019.08.006

**Published:** 2019-08-21

**Authors:** Fabio Medas, Pierpaolo Coni, Francesco Podda, Claudia Salaris, Federico Cappellacci, Gavino Faa, Pietro Giorgio Calò

**Affiliations:** a Department of Surgical Sciences, University of Cagliari, Cittadella Universitaria, SS554, Bivio Sestu, 09042, Monserrato, Italy; b Division of Pathological, University of Cagliari, Cagliari, Italy

**Keywords:** Papillary thyroid carcinoma, Lymph node metastases, Cervical lymphectomy, Real-time PCR, One-step nucleic acid amplification (OSNA)

## Abstract

**Background:**

The incidence of node metastases in papillary thyroid carcinoma (PTC) is high, ranging from 20% to 90%. Prophylactic central lymph node compartment dissection (CLND), suggested from the latest guidelines for high-risk tumors, meets resistance due to the high incidence of postoperative complications. Recently, new molecular biologic techniques, such as One Step Nucleic Acid Amplification (OSNA), have spread widely, allowing to quickly isolate, amplify and quantify mRNA encoding for proteins selectively present in neoplastic cells, as Cytokeratine-19. The aim of this study is to evaluate the application of OSNA to intraoperative diagnosis of node metastases of PTC.

**Methods:**

We included in the study patients with preoperative diagnosis of PTC; from each patient one or more lymph nodes were collected. To assess OSNA accuracy, each lymph node was divided into two halves: the first one was analysed with histopathological and immunohistochemical examination, whereas the second was studied with OSNA.

**Results:**

Twenty-six lymph nodes from 13 patients were included in the study. Overall, OSNA sensitivity was 87.5%, specificity 94.4%, positive predictive value 87.5%, negative predictive value 94.4% and accuracy 92.8%.

**Discussion and conclusion:**

OSNA is effective in detecting lymph node metastases of PTC. Considering the high risk of complications in CLND, and the uncertain prognostic value of lymph node metastases of PTC, OSNA seems to be a promising tool to identify intraoperatively patients who may benefit from CLND.

## Highlights

• Prophylactic central lymph node dissection in patients with differntiated thyroid carcinoma is not universally adopted due to high incidence of postoperative complications.
• One Step Nucleic Acid Amplification (OSNA) allows to quickly isolate, amplify and quantify mRNA encoding for proteins selectively present in neoplastic cells.
• OSNA is effective in diagnosis of lymph node metastases of differentiated thyroid carcinoma.

## 1 Introduction

Papillary thyroid carcinoma (PTC) is the most common malignant thyroid neoplasm; it originates from follicular cells of thyroid gland, and represents over 80% of thyroid tumors. The incidence of PTC has progressively been increasing in the last decades, doubling since the 1970s, due to the diffusion of screening ultrasound [[1], [2], [3]]. Although PTCs are considered slow-growing tumors, the incidence of node metastases is high, ranging from 20% to 90% [1,2,4,5]. The real impact of node metastases on prognosis is still a matter of debate: reports in literature demonstrate a reduction of disease-free survival but are divergent on overall survival [2,6,7]. Diagnostic tools have poor accuracy for central lymph node compartment, which is the most frequent site of metastases.

The last guidelines suggest a prophylactic central lymph node compartment dissection (CLND) in patients with high-risk PTC; nevertheless, this indication meets resistance due to the higher incidence of postoperative complications, especially hypoparathyroidism and recurrent laryngeal nerve (RLN) palsy.

Recently, new molecular biologic techniques have spread widely, mainly in diagnosis of node metastases in breast carcinoma; these assays allow to quickly isolate, amplify and quantify mRNA encoding for proteins selectively present in neoplastic cells, as Cytokeratine-19 (CK-19). One Step Nucleic Acid Amplification (OSNA) is routinely used in diagnosis of node metastasis in sentinel lymph node (SNL) of patients affected from breast cancer. The aim of this study is to evaluate if application of OSNA to intraoperative diagnosis of node metastases of PTC is possible.

## 2 Methods

After receiving approval of Local Ethic Committee, we enrolled in the study patients with preoperative cytological diagnosis of PTC. The patients underwent surgery at our department from May 2016 to May 2018. Neck US was routinely performed preoperatively. We included in the study patients with clinically uninvolved lymph nodes (cN0) or with preoperative or intraoperative findings of node metastases (cN1). Informed consent was obtained from every patient.

**Fig. 1:**
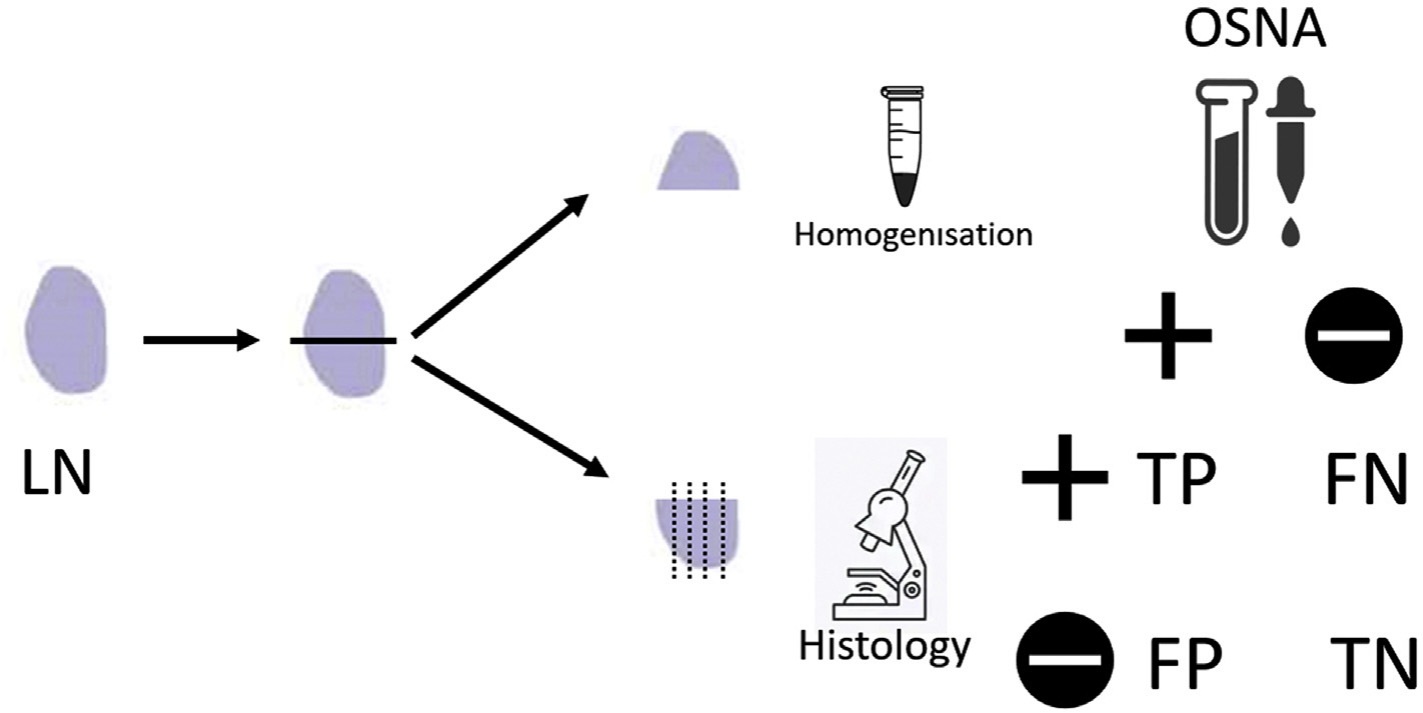
Each collected lymph node (LN) was divided into two halves: the first one was analysed with histopathological and immunohistochemical examination; the second was studied with OSNA after homogenisation of the specimen. Results of the two methods were compared to define true positive (TP), true negative (TN), false positive (FP) and false negative (FN) results.

All the patients underwent total thyroidectomy, associated to CLND and, in case of preoperative studies positive for lateral cervical lymph node metastases, to modified radical lateral neck dissection. From each patient, one or more lymph nodes, among those excised for lymphectomy, were randomly selected and included in the study. Each collected lymph node was weighed and measured, and then divided into two halves: the first one was analysed with histopathological and immunohistochemical examination, whereas the second was studied with OSNA (Fig. 1).

The first half was fixed with formaldehyde; sections were then stained with Haematoxylin-Eosin (H&E). Immunohistochemical analysis was performed with streptavidin-biotin technique on paraffin sections using anti-pan-cytokeratin antibody.

According to manufacturer's instructions (Sysmex, Kobe, Japan), the second half was homogenised in a mRNA stabilizing solution (Lynorhag, Sysmex); the homogenate was briefly centrifugated and directly used as a template for reverse transcription loop-mediated isothermal amplification (RT-LAMP). Amplification of CK-19 mRNA was automatically performed in a RD-100i instrument (Sysmex); results were presented in CK-19 mRNA number of copies/microliter.

Depending on the number of copies/microliter, OSNA test was defined negative (<250 copies/microliter) or positive (≥250 copies/microliter); positive results were then classified as micrometastasis (≥250 and < 5000 copies/microliter) or macrometastasis (≥5000 copies/microliter).

OSNA results were compared to histopathological examination; based on the matching between the techniques, we identified:

- True positive (TP) result: diagnosis of lymph node metastasis with both histopathological and OSNA examinations;
- True negative (TN) result: diagnosis of no lymph node metastasis with both histopathological and OSNA examinations;
- False positive (FP) result: uninvolved lymph node at histopathological examination, with OSNA diagnosis of metastatic lymph node;
- False negative (FN) result: diagnosis of lymph node metastasis at histopathological examination, with negative OSNA exam.

We defined:

Sensitivity: TP Results/(TP Results + FN Results)
Specificity: TN Results/(TN Results + FP Results),
Positive Predictive Value: TP Results/(TP Results + FP Results)
Negative Predictive Value: TN Results/(TN Results + FN Results)
Accuracy: (TP + TN)/(TP + TN + FP + FN).

## 3 Statistical analysis

All data was anonymized and de-identified prior to analysis and the study was reported in line with the STARD (Standards for the Reporting of Diagnostic accuracy studies) criteria and the declaration of Helsinki and registered under www.researchregistry.com. Data were analysed with MedCalc vers. 18.11.3.

## 4 Results

Thirteen patients (1 male and 12 females) with preoperative diagnosis of PTC were included in the study, with a mean age of 37.8 ± 11.8 years. Surgical procedure consisted in total thyroidectomy associated to CLND in 6 (46.2%) patients and to CLND and modified lateral neck dissection in 7 (53.8%) (Table 1). The mean number of sampled lymph nodes from each patient was 2 (range 1–4). Full description of sample site is reported in Table 2. Mean weight of the excised lymph nodes was 220.8 ± 262.6 mg, mean length was 10 ± 3.7 mm and mean height was 5.7 ± 1.7 mm. After division of the samples, the OSNA half mean weight was 113.3 ± 160 mg.

**Table 1:** Demographics and surgical procedure.

	Patients (n = 13)
Sex	
Male	1 (7.7%)
Female	12 (92.3%)
Age, years (mean ± sd)	37.8 ± 11.8
Surgical procedure	
TT + CLND	6 (46.2%)
TT + CLND + LND	7 (53.8%)

TT: Total thyroidectomy; CLND: Central lymph node compartment dissection; LND: lateral neck dissection.

**Table 2:** Features of the samples.

	Lymph nodes (n = 26)
Sampling site	
II level	1 (3.8%)
III level	5 (19.2%)
VI level	10 (38.5%)
VII level	3 (11.6%)
Perithyroid	7 (26.9%)
Total weight of the sample, mg (mean ± sd)	220.8 ± 262.6
Length, mm (mean ± sd)	10 ± 3.7
Height, mm (mean ± sd)	5.7 ± 1.7
Weight of OSNA sample, mg (mean ± sd)	113.3 ± 160

Both histopathological and OSNA examinations revealed metastases in 8 (30.8%) lymph nodes and no metastases in 18 (69.2%); according to the number of copies, 4 cases were classified as micrometastases and 4 as macrometastases. Concordance between the two tests was found in 24 (92.3%) lymph nodes; in one case histopathological examination revealed a node metastasis with OSNA test being negative (false negative test), and in one case histopathological examination was negative with OSNA test being positive (false positive test) (Table 3 and Table 4).

**Table 3:** Results of histopathological examination and OSNA.

	Lymph nodes (n = 26)
Histopathological examination	
Metastatic lymph nodes	8 (30.8%)
Non-metastatic lymph nodes	18 (69.2%)
OSNA	
Positive test	8 (30.8%)
Micrometastasis (mean copies/microliter)	5 (3.2 × 10^3)
Macrometastasis (mean copies/microliter)	3 (1.53 × 10^5)
Negative test	18 (69.2%)
Concordance between diagnostic tests	24 (92.3%)

**Table 4:** Performance of OSNA.

	Lymph nodes (n = 26)
Test result	
True positive	7 (27.9%)
True negative	17 (65.4%)
False positive	1 (3.8%)
False negative	1 (3.8%)
Sensitivity	87.5% (95% CI: 47.3%–99.7%)
Specificity	94.4% (95% CI: 72.7%–99.9%)
Positive predictive value	87.5% (95% CI: 50.6%–97.9%)
Negative predictive value	94.4% (95% CI: 73%–99%)
Accuracy	92.3% (95% CI: 74.9%–99%)

CI: Confidence interval.

Overall, sensitivity was 87.5%, specificity 94.4%, positive predictive value 87.5%, negative predictive value 94.4% and accuracy 92.8%.

## 5 Discussion

Prognostic value of lymph node metastases in PTC is a matter of debate. If some authors have shown that lymph node metastases do not decrease survival rate [[5], [6], [7], [8], [9], [10]], others have suggested that lymph node involvement is a significant prognostic factor affecting both disease-free [11] and overall survival [12,13] rate. Consequently, also the optimal extent of cervical lymphectomy remains controversial.

In the past, lymphectomy was reserved to patients with clinically involved lymph nodes (cN1) and consisted in radical neck dissection.

Nevertheless, the most frequent site of node metastasis is central neck compartment [7,[14], [15], [16], [17]], defined as the anatomical area between the hyoid bone, the suprasternal notch, and the carotid arteries bilaterally. Ultrasound is the gold standard in diagnosis and preoperative evaluation of PTC, but its accuracy in detecting node metastasis of the central compartment is low, due to technical limits because of the presence of air in the trachea which hampers diffusion of ultrasound [1,18].

For these reasons, in the last decade the major guidelines on thyroid cancer have suggested a prophylactic CLND for patients with high-risk PTC and clinically uninvolved lymph nodes (cN0), consisting in excision of peri-thyroidal, pre-cricoid and recurrent lymph nodes [19].

Supporters of this approach suggest that selective lymphadenectomy is not sufficient and is associated with a greater risk of local relapse [11,13,20], thus prophylactic CLND allows to reduce the necessity of second surgery, other than providing a better staging of the tumor [16,[21], [22], [23]].

By the other side, many authors have shown that this approach is associated with higher incidence of transient hypoparathyroidism [6,7,24] and RLN injury [7]; also considering that the real impact of metastatic lymph node on prognosis is doubtful, they discourage prophylactic CLND as routine approach.

In this scenario, finding new diagnostic tools which allow to identify preoperatively or intraoperatively lymph node metastases seems to be of considerable importance.

Sentinel lymph node (SLN) is the first tumor lymphatic drainage station. SLN biopsy permits to assess nodal status and to decide the proper treatment of regional lymph node stations: if SLN is positive, lymphectomy of the affected compartment is performed. This approach is routinely used for breast cancer and, recently, has been proposed also for PTC.

Several methods of intraoperative analysis of SLN in PTC have been tested, including frozen sections and node-Thyroglobulin measurement, resulting in discordant results between intraoperative analysis and definitive histopathological findings, with poor values of sensitivity and specificity [25].

During the last few years, molecular-biology techniques have been introduced to identify tumor-specific markers. Among these, reverse-transcription polymerase chain reaction (RT-PCR) is used to identify tumor-specific mRNA, and is useful to detect lymph node metastases; unfortunately, this method is not suitable for clinical practice, due to its complexity and its time-consuming nature.

The OSNA method was first described by Tsujimoto and colleagues to detect node metastases in breast cancer [26]. This technique allows to quickly isolate, amplify and quantify mRNA encoding for CK-19; a single examination takes 30–40 min, is highly automated, and does not require sterile working conditions: that makes OSNA suitable for intraoperative examination. The excellent accuracy of OSNA in detecting node metastases in breast cancer has been shown in several clinical studies [[26], [27], [28], [29], [30]]. Later, OSNA has been shown effective in detecting lymph node metastases from other types of tumors, such as colorectal carcinoma, endometrial carcinoma, gastric carcinoma, head and neck squamous cell carcinoma and lung carcinoma [[31], [32], [33], [34]].

Chu reported that CK-19 is strongly expressed in cellular membrane of over 70% of papillary thyroid carcinomas [35], and this positivity is also seen in metastatic cells within the lymph node; CK-19 is not present in other elements of the lymph node, thus CK-19 is eligible as a marker of metastasis of PTC.

These considerations have led us to validate OSNA as a diagnostic tool in evaluating lymph node status in PTC. We decided to compare OSNA results with the gold standard diagnostic method represented from histopathological examination.

We analysed 26 lymph nodes from 13 patients; for the purpose of our study, each lymph node was considered as an independent case. Results of OSNA and histopathology were according in 24 (92.3%) cases: 7 (27.9%) lymph nodes were positive both on OSNA and on histopathology examinations, while 17 (65.4%) were negative. We found discording results in 2 (7.7%) cases. In one case OSNA was positive for micrometastasis (number of copies = 2.7 × 103), while histopathological examination didn't find any metastatic cell. We found the opposite result in another lymph node, with histopathological examination demonstrating macrometastasis.

These findings could be explained by the fact that metastatic deposits are often small and irregularly distributed within the lymph node; in our study half of the node was analysed with OSNA and the other half with histopathological examination, thus the same slice cannot be analysed with both the techniques. It's likely that, in discordant cases, metastatic cells were restricted to the half which was analysed with OSNA or histopathology, respectively.

In this regard, it seems important to underline that histopathology examination is performed on each lymph node with a single H&E section; it's possible that the half lymph node analysed with histopathological examination was containing small foci of carcinoma that were not included in the analysed section, resulting in a false positive result. Thus, it's reasonable that the number of positive lymph nodes is underestimated by histopathology.

By the other side, OSNA allows to evaluate the entire lymph node, enabling to detect a greater number of small metastatic foci, as reported for breast cancer from Osako et colleagues [36]. In fact, as already stated, classic histopathological examination is usually performed only on one section of the lymph node, thus small foci of metastasis could be misdiagnosed because they are not included in the analysed section. This fact has been well described from Del Carmen et colleagues, that demonstrated that, in PTC, OSNA led to upstaging from pN0 to pN1 25% of patients with negative lymph nodes assessed by classic pathologic tools [37].

False negative results could also be related to a low expression of CK-19 as it is the only marker used in OSNA assay, as reported for breast carcinoma [30].

It would be very interesting to perform OSNA on the whole lymph node as is done with breast cancer; unfortunately, this was not possible in our study due to legal and ethical reasons because OSNA wasn't still validated as effective in detecting node metastasis in PTC.

Overall, our study demonstrated that OSNA has high accuracy in detecting lymph node metastases of PTC.

To the best of our knowledge, three studies before ours have tried to validate OSNA in PTC [[37], [38], [39]] with a comparison between OSNA and histopathological examination. Results, including ours, are summarized in Table 5. Overall, sensitivity of OSNA ranges from 85% to 90.5%, specificity from 88.9% to 94.4%, positive predictive value from 77.3% to 87.5%, negative predictive value from 92.8% to 94.4%. Concordance rate between OSNA and histopathological examination is high, ranging from 87.7% to 92.3%.

**Table 5:** Summary of the studies that have assessed OSNA accuracy in diagnosis of lymph nodes metastases of papillary thyroid carcinoma.

	N. of lymph nodes	TP	TN	FP	FN	Sensitivity	Specificty	PPV	NPV	Accuracy
Del Carmen	284	84	168	19	13	86.6%	89.8%	81.5%	92.8%	88.7%
Kaczka	65	17	40	5	3	85%	88.9%	77.3%	93%	87.7%
Gonzalez	50	19	26	3	2	90.5%	89.7%	86.3%	92.9%	90%
Medas	26	7	17	1	1	87.5%	94.4%	87.5%	94.4%	92.3%

TP: True positive, TN: True negative, FP: False positive, FN: False negative, PPV: Positive predictive value, NPV: Negative predictive value.

It's interesting to note that, according to our study, also in the other studies positive predictive value is constantly lower than negative predictive value. This finding had already been extensively reported and discussed from Shi et colleagues in a metanalysis on OSNA in breast cancer on 2833 patients [30]. As already discussed, this fact is likely to be related to the lower sensitivity of classic histopathology examination than OSNA.

However, since now OSNA has not been used in clinical practice because all the works were focused on validation of OSNA in thyroid carcinoma. Published studies, including ours, demonstrate that OSNA is effective in diagnosis of node metastasis from PTC, and suggest that OSNA is even more sensitive than histopathological examination. Thus, the next step should be to identify SLN and to analyse the whole lymph node with OSNA: CLND should be reserved to patients with evidence of metastatic SLN. This approach could avoid unnecessary lymphectomy of the central compartment, reducing the incidence of postoperative complications.

Gonzalez et colleagues in a recent study reported a series of 35 patients with PTC which underwent analysis of SLN with OSNA and conventional cytology [40]. In this study 110 SLNs were analysed: conventional cytology was positive in 25 (22.7%) lymph nodes and 14 patients (40%), while OSNA was positive in 42 (38.2%) lymph nodes and 23 patients (65.7%); all the discordant cases were OSNA positive and cytologically negative. This study strongly demonstrates that OSNA is reliable in detecting node metastasis and has greater sensitivity than cytology, even if it should be underlined that accuracy of cytology is considerably lower than histopathology.

By the other side, the high sensitivity of OSNA in detecting metastatic lymph nodes could lead to a greater number of unnecessary lymphectomies, also considering that prognostic value of metastatic lymph nodes in PTC is still unclear.

Another limit of OSNA is that, requiring the analysis of the whole lymph node, histopathological examination is precluded, thus information about extranodal extent is not provided; furthermore, OSNA is able to detect only metastatic CK-19 positive foci, hence CK-19 negative metastatic cells would be miss-diagnosed. In addition, in case of strong suspicion of lymph node metastasis due to clinical features but with negative results of SLN at OSNA, the surgeon would be in difficulty with the decision about the more appropriate treatment, also considering medico-legal implications.

Finally, OSNA is formally able to analyse only lymph nodes of at least 50 mg; this weight limit may be relevant in lymph nodes of the central compartment, which are smaller and lighter than those of the axillary compartment: it is possible that excised lymph node would be lighter than this threshold, and this could raise questions about the reliability of OSNA result.

## 6 Conclusion

Our study confirms that OSNA is effective in detecting lymph node metastases of PTC. Considering the higher risk of complications in CLND, and the uncertain prognostic value of lymph node metastases of PTC, OSNA seems to be a promising tool to identify patients who may benefit from CLND and to perform a “tailored surgery”. Considering the high accuracy of OSNA, further studies should provide analysis of the whole SLN with this method.

## Ethical approval

Ethical approval given by Ethic Committee of Azienda Osperdaliero-Universitaria di Cagliari, Italy (Prot. NP/2016/2494).

## Sources of funding

The project reported in this publication received financial support from “Fondazione di Sardegna”, a no-profit organization (grant 2018.1365). The funding was used for acquisition of goods and services inherent the project, and for Article Processing Charge. “Fondazione di Sardegna” did not influence the work in any way, it was not involved in study design, collection, analysis and interpretation of data, in writing the report and in the decision to submit the article for publication.

## Author contribution

Fabio Medas: Conceptualization, Methodology, Investigation, Original draft writing;

Pierpaolo Coni: Data curation, methodology, original draft writing;

Francesco Podda: Data curation, formal analysis, investigation, writing review;

Claudia Salaris: Investigation, conceptualization, writing review;

Federico Cappellacci: Data curation, formal analysis, writing review;

Gavino Faa: Formal analysis, supervision, writing review;

Pietro Giorgio Calò: Conceptualization, Methodology, Supervision, writing review and editing.

## Conflicts of interest

The authors declare that there is no conflict of interest regarding the publication of this work.

## Research registration number

Name of the registry: Clinicaltrials.gov.

Unique Identifying number or registration ID: NCT03889769.

Hyperlink to the registration (must be publicly accessible): https://clinicaltrials.gov/ct2/show/NCT03889769?term=NCT03889769&cond=Thyroid+Cancer&rank=1.

## Guarantor

Fabio Medas.

Pietro Giorgio Calò.

## Consent

Written informed consent was obtained from the patient for publication of this case report and accompanying images. A copy of the written consent is available for review by the Editor-in-Chief of this journal on request.

## Provenance and peer review

Not commissioned, externally peer reviewed.
